# Hybrid total hip arthroplasty in patients aged over 75: Patient-reported outcomes and complication rates

**DOI:** 10.1016/j.jcot.2026.103353

**Published:** 2026-01-19

**Authors:** Cesare Meschini, Mattia Chirico, Matteo Innocenti, Giovanni Valentini, Paolo Salari, Andrea Baldini

**Affiliations:** a University of Florence, School of Human Health Sciences, Largo Brambilla 3, Florence, 50134, Italy; b Department of Orthopedics and Geriatric Sciences, Catholic University of the Sacred Heart, Rome, Italy; c Department of Clinical Orthopaedics, AOU Careggi, University Hospiptal of Florence, Florence, Italy; d Istituto Fiorentino di Cura e Assistenza, 50139, Florence, Italy

**Keywords:** Arthroplasty, Prosthesis fixation, Hip prosthesis, Dual mobility

## Abstract

**Background:**

As the number of total hip arthroplasties (THA) performed in elderly patients continues to rise, the optimal fixation strategy for individuals over 75 years remains debated. Hybrid constructs, combining a cemented femoral stem with an uncemented acetabular component, may offer a balance between immediate mechanical stability and durable biological fixation. This study aimed to evaluate clinical outcomes, complications, implant survivorship, and patient-reported satisfaction following hybrid THA in patients aged >75 years.

**Methods:**

A retrospective multicenter study was conducted including patients ≥75 years who underwent primary hybrid THA between 2017 and 2023, with a minimum follow-up of 12 months. All procedures were performed using a mini-posterolateral approach within a standardized fast-track perioperative protocol. Patients were further stratified into two subgroups based on acetabular articulation: Group A, treated with a dual-mobility construct, and Group B, treated with a fixed-bearing liner. Outcomes included the Oxford Hip Score (OHS), patient satisfaction, complications, and Kaplan–Meier survivorship analyses using best- and worst-case scenarios.

**Results:**

A total of 642 patients were included (mean age 80.0 ± 4.0 years; 72.6 % female), with a mean follow-up of 39.9 ± 19.4 months. The OHS improved from 22.4 ± 3.3 preoperatively to 42.1 ± 2.8 at final follow-up. Overall satisfaction was high, with 93.5 % of patients reporting a score of 3 or 4 on a 5-point scale. Complication rates were low, including dislocation (1.2 %), periprosthetic fracture (0.5 %), infection (0.2 %), aseptic loosening (0.2 %), and reoperation (1.2 %). Thirty-day readmission was 0.8 %. Overall mortality during follow-up was 6.4 %, with no procedure-related deaths. Implant survivorship was 98.6 % in the best-case and 84.1 % in the worst-case scenario.

**Conclusion:**

Hybrid THA in patients over 75 years provides excellent functional recovery, high satisfaction, and low complication rates, supporting its safety and effectiveness in the elderly population. Further long-term prospective studies are warranted.

## Graphical abstract

**Figure:**
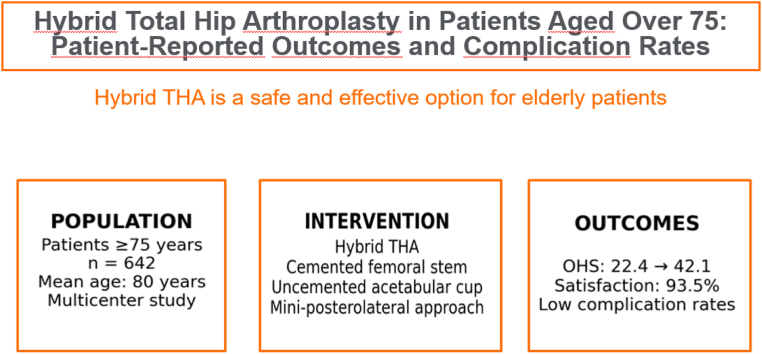

## 1 Introduction

As life expectancy increases worldwide, the number of total hip arthroplasties (THA) performed in elderly patients—especially those over the age of 75—continues to rise. In this age group, the choice of implant fixation method is critical for balancing long-term implant survival, perioperative safety, and patient satisfaction. Given the prevalence of osteoporotic bone and multimorbidity in older adults, cemented femoral fixation has regained popularity due to its predictable performance in poor-quality bone 1, 2, 3. Recent literature has emphasized the evolving understanding and refinement of cementation techniques, advocating for standardized protocols and stem design classification systems that can improve outcomes, especially in the geriatric population ^[^4, 5, 6^]^.

Several studies have shown that cemented femoral stems are associated with significantly lower rates of intraoperative and early postoperative periprosthetic femoral fractures in elderly patients.^7^^,^^8^ Data from the American Joint Replacement Registry revealed that cemented fixation in patients over 65 years significantly reduces the risk of periprosthetic femur fractures compared to cementless stems.^7^ Similarly, long-term registry data from the Australian Orthopaedic Association demonstrated lower revision rates with polished cemented stems compared to commonly used cementless stems, up to 17 years of follow-up.^9^ These findings are supported by recent technical recommendations outlining best practices for cementation, particularly in patients with reduced bone stock.^4^^,^^5^

In contrast, uncemented acetabular components have maintained a leading role even in older patients, particularly due to advances in implant design and surface technology. These improvements have enabled reliable osteointegration and stable fixation, especially when paired with dual mobility (DM) cups, which reduce the risk of dislocation—a major concern in the geriatric population 10, 11, 12.

The combination of a cemented femoral stem and an uncemented acetabular component—commonly referred to as a hybrid construct—has become an increasingly attractive option in THA for the elderly. This strategy aims to harness the primary stability and reduced fracture risk of cemented stems while benefiting from the long-term biological fixation of uncemented cups.^3^^,^^11^^,^^13^ Additionally, the use of DM bearings further improves implant stability and lowers the incidence of dislocation, particularly in high-risk patients.^12^

Despite this theoretical advantage, current literature offers limited data specifically addressing clinical outcomes, complication rates, and patient-reported satisfaction following hybrid THA in patients over 75 years of age. Most existing studies focus on either stem or cup fixation independently, or compare cemented versus cementless fixation, without evaluating the hybrid construct as a distinct approach in this age group.^2^^,^^14^^,^^15^

Therefore, the present study aimed to evaluate the clinical outcomes, complication profile, and patient satisfaction following primary hybrid total hip arthroplasty in patients aged >75 years. This investigation seeks to provide evidence on whether the hybrid construct represents an optimal fixation strategy in this growing and vulnerable population.

## 2 Materials and methods

This research was designed as a retrospective, multicenter observational study based on clinical data retrieved from the orthopedic departments of two hospitals, covering procedures performed between January 2017 and December 2023. The study protocol complied with the ethical standards outlined in the Declaration of Helsinki. The study adhered to the ethical principles established in the Declaration of Helsinki and received approval from the local Ethics Committee (approval code 27764_oss).

Patients were considered eligible if they were 75 years of age or older at the time of primary THA, underwent surgery for either primary or secondary osteoarthritis, and received a hybrid implant configuration consisting of a cemented femoral stem and an uncemented acetabular component. In one institution, dual-mobility constructs were routinely used as the default acetabular option, whereas the second center employed fixed-bearing liners coupled with 32- or 36-mm femoral heads. Only patients with a minimum follow-up of 12 months were included.

Patients were stratified according to the type of acetabular bearing into two subgroups.

Group A included patients receiving a modular dual mobility acetabular component, whereas Group B comprised patients treated with a fixed-bearing liner. A predefined subgroup analysis was performed to compare complication rates and patient-reported outcomes between the two bearing strategies.

Exclusion criteria were: age under 75 years, implantation of an uncemented femoral component, incomplete clinical records, or follow-up duration of less than one year.

All procedures were performed through a mini-posterolateral approach under a standardized fast-track perioperative care protocol. Regional anesthesia (spinal) was routinely administered, ensuring rapid postoperative recovery. The use of intra-articular drains was avoided unless specifically indicated. Patients were mobilized on the day of surgery with assistance from a physiotherapist, initially using two crutches. Discharge was typically planned for postoperative day three, contingent upon achieving two main functional goals: safe ambulation with crutches and independent stair navigation.

Pain control was achieved through a multimodal analgesic regimen, which included acetaminophen, a short-course opioid, and a nonsteroidal anti-inflammatory drug (NSAID). Clinical follow-up focused on both general and implant-related complications, such as dislocation, deep infection, periprosthetic fracture, aseptic loosening, wound complications (including dehiscence), clinically perceived limb length discrepancy, and the need for surgical revision.

Functional status was evaluated using the Oxford Hip Score (OHS)^16^, administered preoperatively and at follow-up visits. Additionally, patient satisfaction was assessed through two measures: a visual analogue scale (VAS)^17^ and a 5-point Likert scale ranging from 0 (very dissatisfied) to 4 (very satisfied).

### 2.1 Statistical analysis

Statistical analysis was performed using SPSS software (Version 26.0, IBM Corp., Armonk, NY, USA).

Continuous variables were assessed for normality and are reported as mean and standard deviation; comparisons between groups were performed using the Student's t-test or the Mann–Whitney *U* test, as appropriate. Categorical variables are presented as absolute numbers and percentages and were compared using the chi-square test or Fisher's exact test when expected cell counts were <5.

Kaplan–Meier survival analysis was used to estimate implant survivorship, with reintervention defined as the primary event in the best-case scenario, and both reintervention and death considered as events in the worst-case scenario. Differences in survivorship between groups were assessed using the log-rank test.

Patients without events at the last follow-up were censored at the time of final evaluation.

All statistical tests were two-tailed, and a p value of <0.05 was considered statistically significant.

Failure was defined as any revision procedure requiring removal or replacement of at least one prosthetic component (femoral stem and/or acetabular component), regardless of the underlying cause (aseptic or septic), and open reduction of a dislocation. Closed reductions, non-surgical treatments, and procedures that did not involve component replacement were not considered failure events.

## 3 Results

A total of 642 patients were included in the analysis (Table 1). The cohort was predominantly female (72.6 %), and the average patient age was 80.03 ± 4.03 years. Right-sided procedures were slightly more common (54.8 %) than left-sided ones (45.2 %). Regarding preoperative health status, 77.6 % of patients were classified as ASA II, while 21 % were ASA III and 1.4 % ASA I. The mean BMI was 26.79 ± 5.78.

**Table 1:** Overall descriptive analysis. Dichotomous and ordinal variables were reported as frequency and percentage. Continuous variables were reported as mean and standard deviation. IFCA: Istituto Fiorentino di Cura e Assistenza; AOUC: Azienda Ospedaliero Universitaria Careggi; ASA: American Society of Anesthesiologists; FUP: Follow-up; BMI: Body Mass Index; VAS: Visual Analogue Scale.

Variables	Frequency	Percentage (%)
Centers		
IFCA	460	71.7
AOUC	182	28.3
Sex		
F	466	72.6
M	176	27.4
Operated side		
Left	290	45.2
Right	352	54.8
ASA		
1	9	1.4
2	498	77.6
3	135	21
Dislocation	8	1.2
Infection	1	0.2
Aseptic loosening	1	0.2
Periprosthetic fracture	3	0.5
Reoperation	8	1.2
Dehiscence	7	1.1
Heterometry>1 cm	11	1.7
Rehospitalization within 30 days	5	0.8
Death	41	6.4
Lost at FUP	26	4
Satisfaction		
1	6	0.9
2	10	1.6
3	154	24
4	446	69.5
	**Mean**	**Standard deviation**
Age	80.03	4.03
BMI	26.79	5.78
Follow-up (in months)	39.9	19.40
Oxford pre	22.37	3.26
Oxford post	42.08	2.75
VAS	89.81	10.92

The mean follow-up duration was 39.9 ± 19.4 months. Functional outcomes improved markedly following surgery, with the Oxford Hip Score increasing from 22.37 ± 3.26 preoperatively to 42.08 ± 2.75 postoperatively. Pain control was satisfactory, as indicated by a mean VAS score of 89.81 ± 10.92. Patient satisfaction was consistently high: 93.5 % of respondents reported a satisfaction score of 3 or 4, with nearly 70 % choosing the highest score. Only 4 % of patients were lost to follow-up.

When stratifying patients according to acetabular bearing type, 460 patients (71.7 %) received a dual mobility cup, while 182 patients (28.3 %) were treated with a fixed-bearing liner.

Subgroup analysis revealed a significantly lower dislocation rate in the dual mobility group compared with the fixed-bearing group (0.7 % vs. 2.75 %, p = 0.045).

No statistically significant differences were observed between groups regarding periprosthetic joint infection, aseptic loosening, periprosthetic fracture, reoperation rate, 30-day readmission, or mortality.

Postoperative Oxford Hip Scores were comparable between groups, whereas pain control and overall patient satisfaction were significantly higher in the dual mobility cohort.

Postoperative complication rates were low. Dislocations occurred in 1.2 % of cases, while infections, aseptic loosening, and wound dehiscence were rare (≤1.1 %). Periprosthetic fractures were reported in 0.5 %, and reoperations in 1.2 % of patients. Limb length discrepancy greater than 1 cm was observed in 1.7 %. The 30-day readmission rate was 0.8 %.

All reoperations (n = 8; 1.2 %) corresponded to revision procedures involving the removal or exchange of at least one prosthetic component and were therefore considered failure events in the survivorship analysis.

Indications for revision included postoperative dislocation requiring component exchange (n = 3), deep periprosthetic joint infection (n = 1), periprosthetic femoral fracture requiring stem revision (n = 3), and aseptic loosening of the acetabular component (n = 1). No additional surgical procedures outside these revision events were performed.

Overall mortality during the follow-up period was 6.4 %, with no deaths related to the index procedure. These data were obtained through institutional hospital records, and that no deaths were recorded as directly related to the index procedure or perioperative complications.

In the present study, two Kaplan–Meier survival curves were generated.

**Fig. 1:**
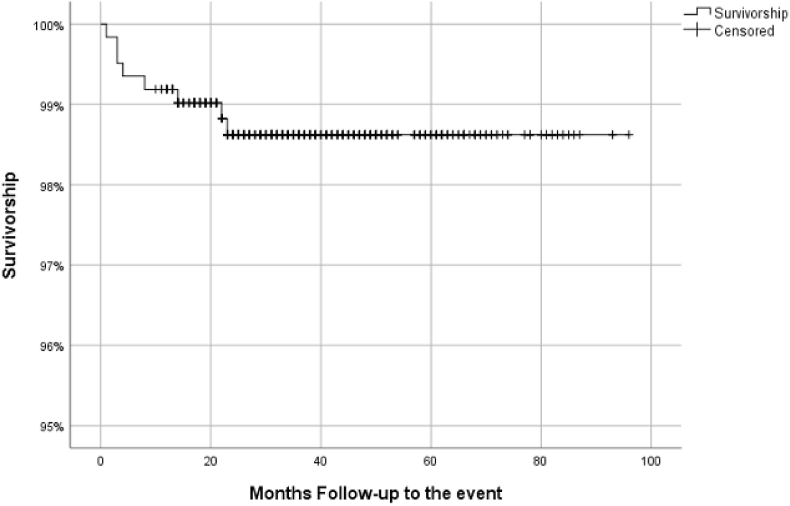
Overall survivorship in best scenario Survivorship: 98.6 % Mean Survivorship: 94.82 % [CI95: 95.63–94.01].

The **first curve** represents the **best-case scenario (**Fig. 1**)**, in which only **reinterventions** were considered as events. All patients from both cohorts were included in the analysis. Censored cases included deaths, losses to follow-up, and patients without events at the final follow-up.

**Fig. 2:**
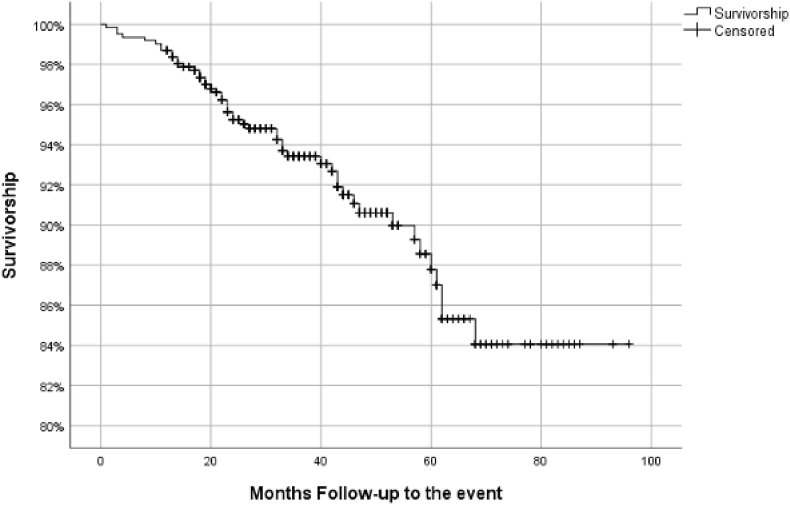
Overall survivorship in worst scenario Survivorship:84.1 % Mean Survivorship: 87.24 % [CI95: 89.65–84.83].

The **second curve** corresponds to the **worst-case scenario (**Fig. 2**)**, in which both **reinterventions and deaths** were considered as failures (events). Additionally, patients lost to follow-up were assigned an estimated failure rate equivalent to that of the overall cohort, in line with the assumption of data missing at random (MAR).

Both curves were used to assess and compare long-term survivorship of the implant.

In the best-case scenario analysis, overall survival estimates remained consistently high over time. At 1 year, the survival estimate was 99.7 %, followed by 99.3 % at 3 years and 98.7 % at 5 years. These results indicate excellent implant survivorship when considering reintervention as the sole event, with minimal decline observed throughout the 5-year follow-up period.

In the worst-case scenario analysis, overall survival estimates showed a gradual decrease over time. At 1 year, the survival estimate was 99.2 %. This declined to 94.1 % at 3 years and further to 88.0 % at 5 years. These figures reflect a more conservative projection of implant survivorship, in which both reinterventions and deaths were considered as events, and losses to follow-up were assigned the expected failure rate of the overall cohort.

## 4 Discussion

This study evaluated the clinical and patient-reported outcomes of hybrid total hip arthroplasty (THA) in patients aged over 75 years, with a focus on complication rates, survivorship, and satisfaction. Our findings support the hybrid construct—cemented femoral stem combined with an uncemented acetabular component—as a safe and effective strategy in this vulnerable population, offering favorable mid-term outcomes and low complication rates.

The observed difference in dislocation rates between dual mobility and fixed-bearing constructs deserves specific consideration. Although the multicenter design introduced heterogeneity in acetabular bearing selection, this allowed a clinically meaningful comparison within a homogeneous hybrid fixation framework.

The significantly lower dislocation rate observed in the dual mobility group supports the growing evidence that dual mobility bearings provide enhanced stability in elderly and high-risk patients, particularly when a posterior approach is used ^12^^,^^13^. Importantly, this benefit was achieved without an increase in other complications, including infection, aseptic loosening, or periprosthetic fracture.

These findings indicate that the favorable outcomes observed in the overall cohort are partly driven by the use of dual mobility bearings, which should therefore be considered a key component of contemporary hybrid THA strategies in elderly patients rather than a secondary or marginal factor.

Our results are consistent with large registry-based studies highlighting the mechanical safety and durability of cemented femoral stems in older adults. Data from major registries, including the American Joint Replacement Registry (AJRR), have consistently shown that cemented fixation significantly reduces the risk of both intraoperative and postoperative femoral fractures in patients over 65 years of age.^7^^,^^18^^,^^19^ These observations are further supported by Brüggemann et al.,^8^ who identified uncemented fixation and advanced age as major risk factors for intraoperative femoral fractures in a national cohort exceeding 200,000 primary THAs.

Within this context, hybrid fixation emerges as a clinically relevant middle ground between fully cemented and fully uncemented THA in elderly patients. In the present cohort, low rates of periprosthetic femoral fracture (0.5 %), dislocation (1.2 %), and revision (∼1.2 %) were observed. Fully cemented constructs have long been considered the reference standard in octogenarians, largely due to their consistently low rates of periprosthetic femoral fracture—reported below 0.5 % in contemporary registry data.^20^^,^^21^ However, these advantages may be counterbalanced by longer operative times and the potential risk of bone cement implantation syndrome in frail patients.^22^

By contrast, fully uncemented THA, although often associated with shorter surgical times, remains burdened by a substantially higher incidence of early periprosthetic femoral fractures in elderly patients with age-related bone loss, with reported rates exceeding 3.5 % in individuals over 75 years.^20^^,^^23^ In our series, the periprosthetic fracture rate was comparable to that of fully cemented stems while avoiding routine acetabular cementation. Likewise, the overall dislocation rate of 1.2 % compares favorably with the 2.1 %–3.8 % reported for fully uncemented THA in similar elderly populations.^21^ Although the selective use of dual-mobility acetabular components may have contributed to this finding, the combination of cemented femoral fixation and uncemented acetabular components appears to provide a balanced strategy optimizing both mechanical stability and biological fixation. These results are in line with previous studies reporting improved survivorship of hybrid constructs compared with fully cemented and fully uncemented fixation in older patients.^24^

Regarding long-term performance, cemented polished femoral stems have demonstrated excellent survivorship. Babazadeh et al.,^9^ analyzing over 200,000 procedures from the Australian registry, reported lower revision rates for cemented stems compared with commonly used uncemented designs up to 17 years postoperatively. Similar long-term outcomes have been described by Firestone et al.^25^ and Park et al.,^26^ particularly with polished, tapered designs. The sustained clinical success of Charnley-type stems, even in osteoporotic bone, further supports the reliability of cemented femoral fixation in elderly patients.^27^^,^^28^

Hybrid fixation leverages the complementary strengths of both fixation principles: immediate mechanical stability on the femoral side and durable biological fixation at the acetabulum. Favorable survivorship and functional outcomes with hybrid constructs have been reported at mid- and long-term follow-up ^24^^,^^25^^,^^28^, ^29^, ^30^. Consistently, our cohort demonstrated substantial functional improvement and high satisfaction, with marked increases in Oxford Hip Scores and the vast majority of patients reporting the highest satisfaction levels. These findings are concordant with prior registry and clinical studies documenting favorable patient-reported outcomes following cemented and hybrid THA in elderly populations.^15^^,^^31^

This study has several limitations that should be acknowledged. First, the multicenter design introduced heterogeneity in acetabular component selection, with different bearing strategies adopted across institutions. Although this reflects real-world clinical practice, it represents a relevant source of variability that may have influenced specific outcomes, particularly instability-related events.

Second, the retrospective nature of the study precluded full control over surgical technique. In particular, details related to cementing technique—an important factor influencing the performance and survivorship of cemented femoral stems—could not be standardized or analyzed in detail across centers.

Although elderly patients are often assumed to have lower functional demands, this factor should be interpreted with caution. In the present cohort, postoperative Oxford Hip Scores reached values consistent with excellent function, suggesting that the high levels of patient satisfaction observed cannot be solely attributed to reduced activity expectations and are more likely reflective of meaningful functional recovery.

Although the use of different acetabular bearings across centers represents a source of heterogeneity, the availability of detailed subgroup data allowed us to directly assess its impact on clinical outcomes, thereby strengthening the robustness and transparency of the present analysis.

Finally, no multivariable regression analysis was performed to identify independent risk factors for complications, as the low number of adverse events limited the statistical power for such analyses.

## 5 Conclusion

In a population of patients aged over 75 years undergoing primary total hip arthroplasty, the hybrid construct combining a cemented femoral stem with a press-fit acetabular component demonstrated excellent mid-term outcomes. The approach was associated with low complication rates, high patient satisfaction, and strong implant survivorship, even under conservative analytical assumptions. Cemented femoral fixation, especially using modern techniques, provided reliable mechanical stability in elderly patients with reduced bone quality, while the use of uncemented cups preserved bone stock and facilitated biological integration. Overall, hybrid fixation represents a safe, effective, and balanced solution for elderly patients, warranting continued use and further investigation in long-term prospective studies.

## Guardian/patient's consent

Informed Consent has been obtained from patient or guardian for the study's participation and publication.

## Author contributions

Conceptualization: C. M., M. C., A.B.; Methodology: G. V., M. C.; Investigation: C. M., M. C., M. I., P. S.; Data Curation: C. M., M. C.; Formal Analysis: M. C.; Writing – Original Draft: C. M., M. C.; Writing – Review & Editing: G. V., A. B.; Supervision: G. V.

## Ethics statement

This study, titled “Hybrid Total Hip Arthroplasty in Patients Aged Over 75: Patient-Reported Outcomes and Complication Rates”, was conducted in accordance with the ethical standards of the institutional and national research committees and with the 1964 Helsinki Declaration and its later amendments.

Ethical approval was obtained from the local Ethics Committee Comitato Etico Regione Toscana.

N° 27764_oss. March 19, 2025.

All participants provided informed consent prior to inclusion in the study. Patient data were anonymized before analysis to ensure confidentiality. No additional interventions, beyond standard clinical practice, were performed for research purposes.

## Funding statement

This research did not receive any specific grant from funding agencies in the public, commercial or not-for-profit sectors.

## Declaration of competing interest

The authors declare that they have no known competing financial interests or personal relationships that could have appeared to influence the work reported in this paper.
